# Electrochemical Reduction of CO_2_ in a Zero‐Gap Electrolyzer Cell on a Metal Molecular Electrocatalyst

**DOI:** 10.1002/open.202400488

**Published:** 2025-06-16

**Authors:** Simphiwe L. Ndlangamandla, Shankara G. Radhakrishnan

**Affiliations:** ^1^ Department of Chemistry University of Pretoria Pretoria 0002 South Africa

**Keywords:** copper, electrochemical CO_2_ reduction, ethylene, polymer electrolyte membrane electrolysis, porphyrins, zero‐gap electrolyzers

## Abstract

Electrochemical reduction of CO_2_ (ECR) on transition metal‐containing porphyrin systems often leads to carbon monoxide. Herein, a modified zero‐gap polymer electrolyte membrane water electrolyzer is used with 5,10,15,20–tetraphenyl–21H,23H–porphine copper (II) as a cathodic electrocatalyst for ECR in 0.1 M NaOH as an aqueous catholyte. The setup yields CO, CH_4_, and C_2_H_4_ along with hydrogen evolution, with selectivity toward C_2_H_4_ formation as indicated by gas chromatographic analysis. Although hydrogen formation is predominant, the system yields a high average current density of 146.94 mA cm^−2^ and a Tafel slope of ≈226 mV dec^−1^ in concurrence. The cyclic voltammetric experiments show the stepwise formation of Cu (II) → Cu (I) → Cu (0) based on the potentials referenced against the reversible hydrogen electrode, which could have been the driving factor for the ECR.

## Graphical abstract

A zero‐gap proton exchange membrane electrolyzer cell using Nafion®117 membrane‐containing copper porphyrin cathode electrocatalyst in CO_2_ saturated 0.1 M aqueous NaOH catholyte produces C_2_H_4_, CO, and CH_4_ as products.

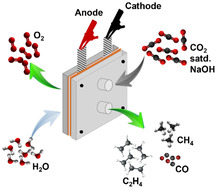

## 1 Introduction

One of the greatest environmental threats of the 21st century is the continuous accumulation of CO_2_ in the atmosphere, which has surpassed the upper limit of 350 ppm, leading to extreme climate changes. In efforts to mitigate CO_2_ footprint, several research approaches have been proposed electrochemical CO_2_ reduction (ECR) that involves coelectrolysis of CO_2_ and H_2_O, being one such approach to produce useful energy dense products, which falls under the carbon capture and utilization technology. These chemicals can be used as fuels or as starting materials for further processing into other value products. Hori^[^ ^1^ ^]^ and several other researchers have widely published in this field, and many review articles are available for reference.^[^ ^2^, ^3^, ^4^ ^]^

ECR is a lucrative approach because i) electrolysis can be performed at room temperature, ii) the safety concerns involving using a hydrogen (H_2_) gas feed are avoided as H_2_ is generated in situ via water electrolysis, and iii) it can be paired with renewable energy sources to convert intermittent electrical energy into stable chemical energy in the form of valuable products. Parameters such as electrocatalyst design,^[^ ^5^, ^6^, ^7^ ^]^ temperature, pH,^[^ ^8^ ^]^ nature of the electrolyte,^[^ ^9^, ^10^ ^]^ electrode composition,^[^ ^11^ ^]^ electrolyzer design,^[^ ^12^, ^13^ ^]^ and applied potential^[^ ^14^ ^]^ can all influence the nature and distribution of ECR products. The applied potential is one of the major parameters through which the distribution of ECR products is controlled. These parameters can be tuned to enhance selectivity and efficiency.^[^ ^15^ ^]^

Since electrocatalysis occurs at the electrode surface, it is important for the catalyst to possess a larger surface area to volume ratio, as it is expected to impact the number of available sites for reaction. Yu et al. recently reported on the design aspects of ECR nitrogen‐containing electrocatalysts based on Marcus's theory.^[^ ^16^ ^]^ In this regard, single‐atom catalysts are gaining prominence as they are good contenders for the judicious use of metals.^[^ ^17^ ^]^ In this, four‐membered nitrogen‐containing (N4) systems, such as phthalocyanines and porphyrins, have gained considerable popularity, as the nitrogen atoms can act as good donors that anchor transition metals to form stable macrocycles (M‐N4 sites). They have been widely studied systems for electrocatalysis and light harvesting.^[^ ^17^, ^18^ ^]^ In particular, porphyrins have made their way into the ECR owing; i) to their excellent solubility in a variety of organic solvents and aqueous medium allowing them to be used in both homogeneous and heterogeneous electrocatalysis,^[^ ^19^ ^]^ ii) to their versatile structure and ease of modification via organic approaches, they can be easily tuned to enhance ECR selectivity for desired products, and iii) to their ability to bind CO_2_ and CO_2_ intermediates thus decreasing the activation potential required for ECR.^[^ ^20^ ^]^

Among the metals from the periodic table, copper and copper‐derived catalysts are the only systems that convert CO_2_ to a range of C_1_ and C_2+_ products owing to their position at the Sabatier maximum on the volcano plot.^[^ ^21^ ^]^ Also, it has been noted that copper‐based electrocatalysts were efficient in promoting ECR under aqueous conditions.^[^ ^22^ ^]^ A variety of literature is available on copper‐based catalytic systems for ECR.^[^ ^19^, ^23^ ^]^

Myriad cell configurations have been used to understand/ improve the efficiency of ECR.^[^ ^12^ ^]^ Ion exchange membrane cells allow for a sophisticated electrolyzer configuration that borrows from the two‐chamber design of the proton exchange membrane (PEM) water electrolyzer to incorporate CO_2_ gas. The central feature is the solid polymer electrolyte separating the electrodes, which allows for a zero‐gap design, improved resistance, better mass transport, and scalability.^[^ ^12^, ^24^ ^]^ Nafion 117, a PEM, is a popular choice because of its acidic nature, which allows for good proton conduction and mechanical properties. Therefore, it allows for migration of protons from the anode to the cathode for H_2_ production, which can be used in situ as a feedstock with CO_2_ gas for ECR. On the other hand, anion exchange membranes are commonly used in ECR, the major disadvantage being the crossover of CO_2_ to the anode, which reduces its availability at the cathode. Thus, the major advantage of PEM is the inhibition of anion conduction and CO_2_ crossover, which can help with the ECR in aqueous media where the solubility of CO_2_ is already a mere 33 mM.^[^ ^24^ ^]^ Taking all of these factors into concert, we have attempted to study the behavior of 5,10,15,20–tetraphenyl–21H,23H–porphine copper (II), simply abbreviated as copper porphyrin (CuP), as a cathodic electrocatalyst for ECR on Nafion membrane with 0.1 m NaOH as a supporting electrolyte. This is the first instance to our knowledge where a zero‐gap electrolyzer that was designed and optimized for water electrolysis was successfully modified for ECR to hydrocarbons using CuP.

## 2 Results and Discussion

**Figure 1:**
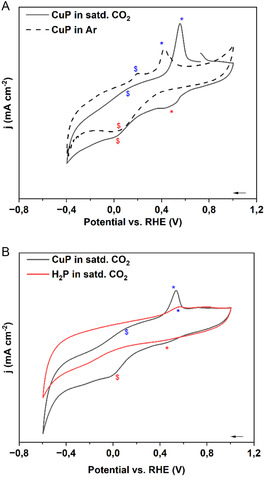
Cyclic voltammograms of A) CuP in 0.1 M aqueous NaOH electrolyte solution; B) H_2_P and CuP in 0.1 M aqueous NaOH saturated with CO_2_. In both cases, a glassy carbon electrode was coated with catalyst ink as described in the Supporting Information.

The cyclic voltammograms of the porphyrins as performed in an argon (Ar)‐bubbled and CO_2_‐saturated aqueous 0.1 m NaOH can be visualized in **Figure** **1**. Initially, the 0.1 M NaOH aqueous solution had a pH of 12.8, which upon saturation with CO_2_ decreased to a pH of 6.9. Figure 1A shows a sharp wave at about 0.42 V versus reversible hydrogen electrode (RHE) in Ar‐bubbled solution marked by a blue asterisk, followed by another feature, indicated by the peso symbol at about 0.2 V versus RHE, and its corresponding reoxidation features indicated in red symbols at 0.05 V versus RHE. In the saturated CO_2_ solution, the reduction features of CuP are shifted more positive, i.e., from 0.42 V to 0.55 V and from 0.2 to 0.012 V versus RHE, and the reoxidation peaks to more negative values, 0.05–0.02 V versus RHE. When the cyclic voltammetry (CV) of CuP in CO_2_ is compared with its metal‐free H_2_P, one can note in Figure 1B that there is a weak feature at about 0.56 V versus RHE. It must be noted that both Cu and porphyrin are active in the potential window probed here.^[^ ^25^ ^]^ Thus, the feature at ≈0.55 V for both CuP and H_2_P can be attributed to both the Cu metal and the porphyrin backbone.

However, the rest of the other features seen for CuP are absent in H_2_P. This lets us assign the peaks at 0.42 and 0.2 V to Cu(II) → Cu(I) and to Cu(I) → Cu(0) reductions, respectively. This is in accordance with the literature for CuP.^[^ ^26^ ^]^ Shifts in reduction peaks to more positive values and the oxidation peaks to more negative values, as noted above, show the readiness of Cu to be coordinated to CO_2,_ supported by the porphyrin backbone. It is also worth noting here that the redox features Cu (II) → Cu (I) → Cu (0) all appear in the presence of dissolved CO_2_, especially Cu (I) → Cu (0). This matches well with the finding by Xu et al.^[^ ^27^ ^]^ who indicate that the co‐operation of Cu(I) and Cu(0) in catalysis can give a good product yield. This lets us conclude that the conditions set out here with CO_2_‐saturated aqueous 0.1 M NaOH are conducive to the ECR on CuP. This result also reinforces the importance of the central Cu (II) atom for enhanced ECR activity.

**Figure 2:**
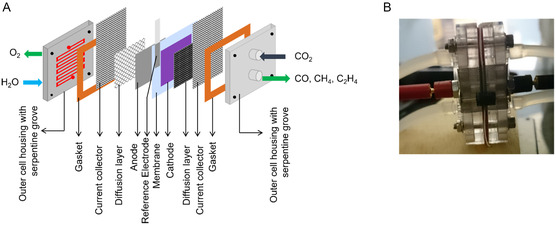
A) A 3D cartoon and B) actual view of the polymer electrolyte membrane zero‐gap electrolysis cell used in this study.

With the above results, we turned to the construction of an electrolyzer cell for the ECR on CuP. **Figure** **2**A shows the cartoon of our electrolyzer wherein the anode electrocatalyst was a conventional 70:30% TaC:IrO_2_ in conjunction with the literature.^[^ ^28^ ^]^ The cathode electrocatalyst was CuP or H_2_P with a loading of 5 mg cm^2^ onto the Nafion 117 membrane, and the method is described in the supporting information.

Both anode and cathode electrocatalysts were spray‐coated directly onto a pretreated Nafion 117 membrane. Thus, the order of layers in the cell is exactly as shown in Figure 2A. The gas diffusion layer at the anode side was a titanium mesh, and this is important because carbon materials may convert the evolved oxygen at the anode into CO_2_.

On the cathode side, carbon paper was used as a gas diffusion layer, and all of these, together with the current collectors and appropriate gaskets, were assembled with a housing containing a serpentine flow‐field design to give an electrolyzer setup as shown in Figure 2B. To easily reference the applied potentials while maintaining a zero‐gap, an Ag/AgCl strip was placed on the cathode side close to the active area, while not touching it. Once assembled, the cell was thoroughly checked for any short circuit before starting the electrolysis. While the anolyte was Type‐1 water, the catholyte solution was 0.1 M aqueous NaOH, and the measured pH was 6.9 after CO_2_ saturation. Both sides were initially allowed to flood with respective solutions, and once flooded, they were kept connected to the closed leak‐free wells, and the electrolysis was performed.

Linear sweep voltammetry (LSV) measurements using the CuP electrocatalyst in the PEM cell display significantly higher current densities under ECR conditions at −1.2 V and an onset potential of about −0.65 V versus RHE. This indicates that the CuP catalyst displays more favorable charge transfer kinetics under ECR conditions than under inert conditions (Figure S1, Supporting Information), as against the H_2_P, where Cu metal is absent. This is also in corroboration with Hori et al, who indicated an onset of CO_2_ reduction at −0.63 V versus RHE.^[^ ^29^ ^]^ In constant voltage electrolysis (chronoamperometry (CA)), the current density reached an average of 146.94 mA cm^−2^ over two hours, while within the first five minutes, the current density reached 202.4 mA cm^−2^. The headspace sampled at the fifth minute from CA and analyzed with gas chromatography (GC) revealed the formation of carbon monoxide (CO), methane (CH_4_), and ethylene (C_2_H_4_). In literature, it has been shown that, at low concentrations of KOH as an electrolyte and low flow rates, C_2_H_4_ is favored.^[^ ^12^, ^30^ ^]^ This was indeed the case in our setup, which uses NaOH as the electrolyte.

The Faradaic efficiencies (FE) were calculated as per the equation below.^[^ ^31^ ^]^

(1) $$\text{FE}=\;\frac{n}{Q/ZF}=\frac{nZF}{Q}$$

where *n* is the number of moles of the generated product, mol; *Q* is the total number of electrons passed during electrolysis in Coulombs, C; *F* is Faraday's constant (96,485 C mol^−1^); and *Z* is the number of electrons required to get one molecule of the resultant product. The reactions leading to the formation of the quantified products are shown in **Table** **1**.^[^ ^23^ ^]^

**Table 1:** Electrochemical reactions of the products of electrochemical CO_2_ reduction^[^ ^23^ ^]^ at the cathode with equilibrium potentials obtained in this study.

Reaction	*E* ^o^/(V vs RHE)	Product
2H_2_O → O_2_ + 4 H^+^ + 4e^−^	+1.23	Oxygen evolution reaction
2 H^+^ + 2e^−^ → H_2_	0.00	HER
CO_2_ + 2 H^+^ + 2e^−^ → CO_(g)_ + H_2_O	–0.10	Carbon monoxide (CO)
CO_2_ + 8 H^+^ + 8e^−^ → CH_4 (g)_ + 2H_2_O	+0.17	Methane (CH_4_)
CO_2_ + 12 H^+^ + 12e^−^ → C_2_H_4 (g)_ + 4H_2_O	+0.08	Ethylene (C_2_H_4_)

**Figure 3:**
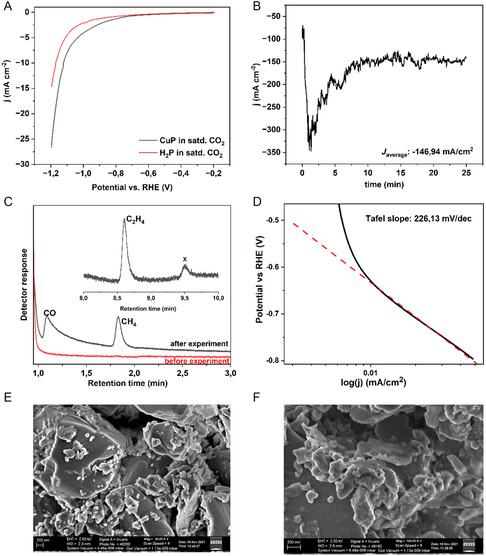
A) Linear sweep voltammograms of CuP and H2P acquired at a scan rate of 10 mV sec^−1^, B) constant voltage electrolysis of CuP at −1.2 V versus RHE, C) gas chromatographic profiles of the head space sampled during the electrolysis of the zero‐gap cell containing 0.1 M aqueous NaOH as a catholyte saturated with CO_2_. The feature marked ‘x’ was also seen in a blank CO_2_ sample before the electrolysis, and hence, not of value. D) Tafel slope analysis for the CuP profile. E) Preelectrolysis SEM image of the catalyst ink containing CuP. F) Postelectrolysis SEM image of the catalyst ink containing CuP.

Although the current densities were high, the FEs were found to be 0.103%, 0.155%, and 2.17% for CO, CH_4_, and C_2_H_4_, respectively, accompanied by H_2_ production. One could attribute these low FEs to a decrease in the local pH at the electrode surface during electrolysis. As the reaction progresses, CO_2_ gets depleted, and the acidic nature of the Nafion membrane could have affected the local pH, and the hydrogen evolution reaction (HER) was favored over ECR. Pre‐ and postelectrolysis characterization of the catalyst surface using scanning electron microscopy (SEM) (**Figure** **3**E,F) showed that the catalyst surface was not completely degraded, which is indicative of the catalyst stability. Furthermore, as the CO_2_ was being used up during the reaction, depletion of dissolved CO_2_ with time was unavoidable, as the system was stagnant, and prolonged electrolysis could not be performed. Even though the system was stagnant, the electrode–electrolyte interface will remain dynamic, as the gas bubbles of the products formed will keep evolving off the surface.^[^ ^32^ ^]^ This was indeed the case, where about 10 mL of gas was collected over a time period of 400 s (6.7 min), which translates to about 2.5 mL min^−1^ of product mix formed. Thus, this is a very positive result considering the conditions, including the solubility of CO_2_ being very low, 33 mM in water.^[^ ^33^ ^]^ To understand the mechanism of C_2_H_4_ formation, we performed Tafel analysis, and the slopes were found to be ≈226 mV dec^−1^ (Figure 3D). This is similar to literature values,^[^ ^34^ ^]^ where Tafel slopes of 255 mV dec^−1^ were obtained, which was attributed to CO_2_ delivery and product desorption from the electrocatalyst surface. In the presented work, the reason could have been the former CO_2_ delivery to the electrocatalyst surface. Further, when CO_2_^−•^ is the rate‐determining step, the Tafel slopes were reported to be 118 mV dec. Kastlunger et al.^[^ ^8^ ^]^ had performed a theoretical analysis corroborating their experiments, wherein, when C_2+_ products were formed on Cu‐based electrocatalysts during ECR, the dimerization of *CO is favored over initial protonation to form intermediates such as *CHO/*COH. It was found experimentally that at low pH, where H_3_O^+^ is the proton donor, CO–CO dimerization is the rate‐determining step, thus, confirming *OCCO as the intermediate species in the pathway to C_2_H_4_ formation. In our study, an indirect confirmation can be derived from the detection of CO as one of the products with GC analysis, which provided a clue as to what might have happened at the electrode surface. The mechanism typically could have involved the dimerization of two *CO molecules at the electrocatalyst surface, which led us to deduce that the *OCCO dimer must have been formed, leading to C_2_H_4_ as a major product. Jiang et al. also reported Tafel slopes of about 211 mV dec^−1^ for C_2_H_4_ formation on CuO‐based catalysts.^[^ ^35^ ^]^ Further, Limaye et al. note that the rate‐determining step and the product formation will have to happen with dependence on the potential at which the intermediate forms exist in equilibrium. Therefore, higher Tafel slopes translate to the assignment of the initial rate‐determining step and are not limited to the “cardinal” values of 60 or 120 mV dec^−1^.^[^ ^8^, ^36^ ^]^

In bridging lab‐scale research to fab‐scale, one needs to calculate the overall energy efficiency of the ECR among many other benchmark parameters.^[^ ^37^ ^]^ To calculate the overall energy efficiency, the anodic reaction potential must be considered; however, it is commonly omitted in the fundamental studies,^[^ ^38^ ^]^ which is also the case in our study, as the aim of this work was to modify an off‐the‐shelf water electrolyzer cell to be used for ECR.

## 3 Conclusion

In the current study, we have been able to demonstrate that it is possible to modify an off‐the‐shelf, compact PEM water electrolyzer into an ECR electrolyzer by replacing the internal components with in‐house prepared materials to achieve a zero‐gap electrolyzer cell configuration. Thus, it has been shown that that ECR to hydrocarbons can be achieved with pure water as a proton source at the anode and copper porphyrin as a cathode electrocatalyst in 0.1 M aqueous NaOH electrolyte under stagnant electrolysis conditions produced mixture of gases but with extremely low Faradaic efficiencies of gases 0.103%, 0.155%, and 2.17% for CO, CH_4_, and C_2_H_4_, respectively, accompanied by H_2_ production at ambient conditions. In spite of obtaining low FEs, the study shows a promise with high current density of up to 202.4 mA cm^−2^ at and an average current density of 146.94 mA cm^−2^ the end of two‐hour constant potential electrolysis. A Tafel slope of about 226 mV dec^−1^ indicated that the formation of C_2_H_4_ must be predominant among other gases, which was indeed the case. Evidence from CV studies indicated a stepwise formation of Cu (II) → Cu (I) → Cu (0) based on the potentials referenced against the RHE at pH 6.9. All of the above studies hint at this system being superior and open doors to a zero‐gap electrolyzer setup using PEM water electrolysis, leading to ECR to useful products. A summary of various other publications where ECR to hydrocarbons was achieved using Cu (II) porphyrins can be found tabulated in the supporting information (Table S1, Supporting Information). No further studies were undertaken in this work as the electrolyzer configuration and the nature of the electrolyte were the main focus. Important aspects that will form the basis of further studies can be maintaining a flow rate in this system to mitigate the CO_2_ depletion at the electrocatalyst surface, studying the cell voltage, and thereby calculating the energy efficiency all of which can help comparing the ethylene production from lab scale to industrial benchmarks and shine light on the challenges faced by the use of zero‐gap CO_2_ electrolyzer in converting CO_2_ to ethylene at higher FEs.

## 4 Experimental Section

Two types of electrochemical experiments were performed on the catalysts: a) a regular CV to understand the redox behavior of the electrocatalysts and b) regular CO_2_ electrolysis in a zero‐gap electrolyzer set up with the CuP as a cathode electrocatalyst which is the focus of this work and IrO_2_ supported on TaC (70:30%) as an anode electrocatalyst both coated on each side of pretreated Nafion 117 solid electrolyte membrane. A detailed description of the Nafion pretreatment and the spray coating is described in the supplementary section.

As a control experiment, 5,10,15,20–tetraphenyl–21H,23H–porphine or meso–tetraphenylporphyrin (H_2_P) was subjected to the same experiments that were performed on 5,10,15,20–tetraphenyl–21H,23H–porphine copper (II) (CuP). The electrochemical methods involved CV, LSV, and CA. The products obtained from the CO_2_ electrolysis were analyzed using GC.

All the potentials were converted from the saturated Ag/AgCl reference electrode to a RHE using the expression

(2) $$E_{\left(\text{vs}.\text{ RHE}\right)}=E_{\left(\text{vs}.\text{ Ag}/\text{AgCl}\right)}+0.197\text{ V}+0.0591\times \text{pH}$$

Further experimental details can be found in Supporting Information.

## Conflict of Interest

The authors declare no conflict of interest.

## Author Contributions

**Simphiwe L. Ndlangamandla**: conceptualization (equal); formal analysis (equal); methodology (equal); writing—original draft (lead). **Shankara G. Radhakrishnan**: conceptualization (equal); formal analysis (equal); funding acquisition (lead); methodology (equal); resources (lead); supervision (lead); writing—review & editing (lead).

## Supporting information

Supplementary Material

## Data Availability

The data that support the findings of this study are available from the corresponding author upon reasonable request.
