# Supplementary material for: Electrochemical Reduction of CO2 in a Zero‐Gap Electrolyzer Cell on a Metal Molecular Electrocatalyst
Source: ChemistryOpen. 2025 Jun 16;14(8):e202400488. doi: 10.1002/open.202400488 (PMC12368890; doi:10.1002/open.202400488)
Supplement: Supplementary file 1 — Supplementary Material [file OPEN-14-e202400488-s001.pdf]

# Supporting Information

## Electrochemical reduction of $CO_2$ in a zero-gap electrolyser cell on a metal molecular electrocatalyst

Simphiwe L Ndlangamandla and Shankara G Radhakrishnan*

Department of Chemistry, University of Pretoria, Pretoria 0002, South Africa

Email: u13181867@tuks.co.za; *shankara.radhakrishnan@up.ac.za

**Experimental details / Methods**

**S1. Materials and reagents**

All the solutions/inks were prepared in Type-I water of resistivity >18 MΩ-cm (Merck Milli-Q purification system). All the chemicals and solvents purchased were of the highest purity and/or HPLC grade and used without further purification.

5,10,15,20–tetraphenyl–21H,23H–porphine or meso–tetraphenylporphyrin / ($H_2P$) and 5,10,15,20–tetraphenyl–21H,23H–porphine copper (II) / (CuP) (≥ 99 % pure) were purchased from Sigma-Aldrich, South Africa. Tantalum carbide (TaC, ≥5 μm, 99%), iso-propanol, ethanol, ethylene glycol, $NaNO_3$, NaOH, $H_2SO_4$ (95-97 %) were all purchased from Sigma-Aldrich, South Africa. Hexachloroiridic acid ($H_2IrCl_6{\cdot}4H_2O$; 99%, Ir 38-42 %) was purchased from the SA Precious Metals, South Africa. $H_2O_2$ was (35% w/w aq. stabilized solution) was purchased from Alfa Aesar, South Africa.

Nafion®-117, the proton exchange membrane (PEM), Nafion® perfluorinated resin solution (5 wt % in lower aliphatic alcohols and water), Titanium mesh, and Carbon paper (Toray-H-060, wet proofed), were all purchased from the Fuel Cell Store, USA. The gases $CO_2$ (99.0%) and Argon (high purity) were procured from African Oxygen (Pty) Limited / Afrox, South Africa.

**S1.1 $IrO_2$: TaC (70:30) anode electrocatalyst powder preparation**

The Iridium oxide ($IrO_2$) anode electrocatalyst powder was prepared in-house using a modified version of the Adams fusion method published by Polonsky et al[39], using TaC as a stable electrocatalyst support.

$$H_2IrCl_6 + 6NaNO_3 \rightarrow 6NaCl + Ir(NO_3)_4 + 2HNO_3 \qquad \ldots(1)$$

$$Ir(NO_3)_4 \rightarrow IrO_2 + 4NO_2 + O_2 \qquad \ldots(2)$$

To obtain the anodic water splitting electrocatalyst, 70:30 % TaC: $IrO_2$, an appropriately calculated amount of $H_2IrCl_6{\cdot}4H_2O$ and TaC were weighed into a porcelain crucible along with 10 ml of isopropanol and stirred for an hour. Thereafter, $NaNO_3$ was added to the mixture in 16.7x molar excess. This was then heated to 110 $^{o}$C and stirred until a brown sludge was formed. The crucible was closed with a lid and placed into a Carbolite® furnace and heated to 500 $^{o}$C for two hours, after which it was taken out and allowed to cool to room temperature. The resultant black powder was scraped off from the crucible into a centrifuge tube filled with 6 ml of Type-1 water and centrifuged 4 times, each time discarding the centrifugate and replacing it with 6 ml of Type-1 water between each centrifugation. The remaining black pellet was then dried under vacuum for an hour using a Millipore® filter paper and then allowed to air dry overnight. The electrocatalyst powder was then carefully removed from the Millipore® filter paper into a clean vial.

## S1.2 Nafion® Membrane Pretreatment

Nafion® membrane was cut into a 4 cm x 4 cm square and placed into 60 mL of 3% $H_2O_2$ solution and lightly boiled for 1 hour. The membrane was then washed with Type-1 water and then placed into a beaker containing 60 mL of Type-1 water which was heated to a light boil for 2 hours. The resulting membrane was then placed into 60 mL of 0.5 M sulfuric acid solution and lightly boiled for 1 hour before being stored in Type-1 water until further use.

## S1.3 Reference electrode, Ag/AgCl preparation

The silver strip used in the zero-gap electrolyser assembly (Figure 2, main article) was electroplated with AgCl in a saturated KCl solution using a 9 V battery. The thus formed Ag/AgCl pseudo reference electrode's potential was checked before and after electrolysis for its integrity against a saturated calomel electrode.

## S1.4 Membrane Coating

CuP or $H_2P$ and 70:30 % TaC: $IrO_2$ electrocatalyst inks were prepared by dissolving the appropriate powders in isopropyl alcohol, 5 wt% Nafion® perfluorinated resin solution, and ethylene glycol, sonicating for 30 minutes after each component addition. The inks were then directly spray-coated on a 1 $cm^2$ area of a pre-treated Nafion® membrane using a MAC AFRIC high precision Professional Airbrush, Adendorff Machinery Mart, South Africa. Initially, one side of the electrode was spray-coated and allowed to dry before the other side was spray-coated. to afford a loading of 5 $mg/cm^2$ at the anode and the cathode sides, respectively. After

coating, the catalyst-coated membrane was boiled in 100 mL of 0.5 M $H_2SO_4$ for 2 hours to activate the membrane.

## S1.4 Cell Assembly

A titanium mesh and carbon paper (1 x 1 cm each) were used as anodic and cathodic gas diffusion layers, respectively. The electrolyser cell was then assembled with the cell casing of a commercial demonstrator electrolyser cell (H-Tec, Electrolyser Cell 5, Fuel Cell Store, USA) containing a serpentine flow field using our in-house-made membrane electrode assembly (prepared as described in section 1.4 above). The complete description of the zero-gap assembly can be found in the main article. The cell was then tested for leaks and shorts using a multimeter before running experiments.

## S2 Electrochemical Experiments

All the electrochemical experiments on the cathodic electrocatalyst was performed in 0.1 M aqueous NaOH electrolyte. All the electrolyte solutions were adequately purged with Ar before every experiment. For $CO_2$ involved experiments, the electrolyte was purged with $CO_2$ for more than 2 hours. pH of 0.1 M NaOH prepared in Type 1 water was measured (using Metrohm 780 pH meter) to be 12.8 and after purging it was 6.9. All experiments were performed in a tightly closed, gas leak-free environment with glassware developed in our laboratory.

All the voltammetric experiments – CV, LSV, and constant potential electrolysis (CA) were performed using a PGStat (Autolab, AUT72638, Metrohm AG, South Africa) controlled by Nova® 2.1 software.

## S2.1 Cyclic Voltammetry

CV characteristics of both $H_2P$ and CuP were performed on a standard three-electrode glass cell containing a glassy carbon working electrode, a platinum wire counter electrode, and a saturated KCl, Ag/AgCl electrode. The working electrode was modified with CuP or $H_2P$ catalyst ink, and CV was run in a 0.1 M aqueous NaOH solution, both in the presence and absence of $CO_2$ as described above.

The electrocatalyst ink for CV experiments was prepared by suspending 5 mg of CuP or $H_2P$ in isopropyl alcohol, Type-1 water, and 5 wt% Nafion® perfluorinated resin solution and

sonicating for 30 minutes. The respective ink was then drop-cast onto a polished glassy carbon electrode ($\Phi$ = 3 mm) and allowed to dry under an argon atmosphere.

CVs were performed by sweeping the electrode potential against an Ag/AgCl reference electrode at a scan rate of 10 mV/s and converted to reversible hydrogen electrode (RHE) as described by equation 1 in the main manuscript.

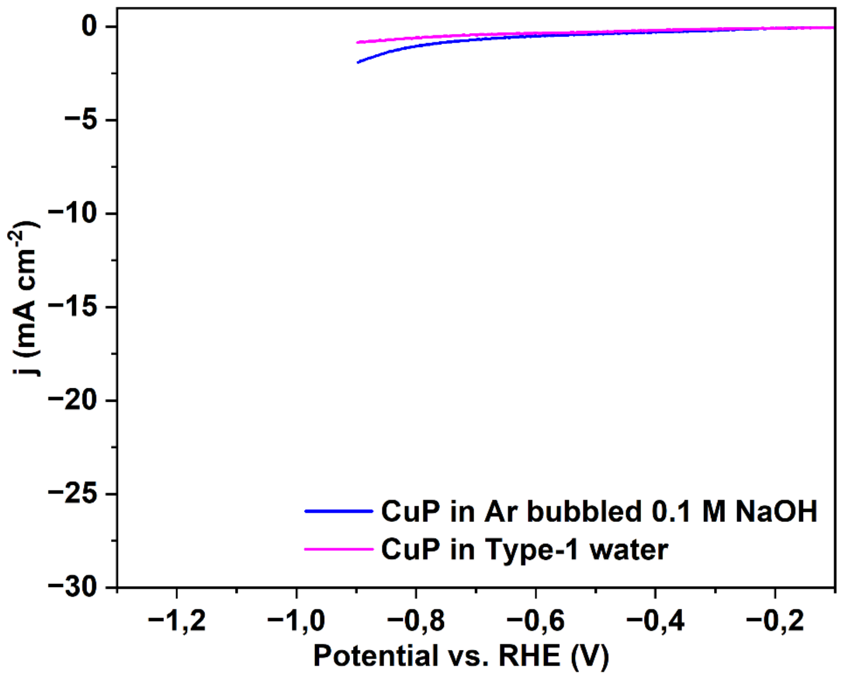


**Figure S1**. Linear sweep voltammograms of CuP in argon bubbled 0.1 M aqueous NaOH and in plain Type-1 water ran at a scan rate of 10 mV/sec. The scale of the graph is kept the same as Figure 3A in main article to show the absence of any activity without $CO_2$.

### S2.2 Electrolysis on the zero-gap electrolyser

The linear sweep voltammograms at a scan rate of 10 mV/s and the constant voltage electrolysis experiments were individually performed on Ar bubbled and $CO_2$ saturated feed into the cathode side by sweeping the electrode potential against pseudo-Ag/AgCl reference electrode and converted to reversible hydrogen electrode (RHE) as described by equation 1 in the main article. The anode was always fed with Type-1 water through a stagnant well.

### S2.3 ECR product characterisation

Manual injection gas chromatography (Agilent 8860 GC-FID/TCD) was used for the ECR gas product characterization and quantification. No liquid products were detected. The

hydrocarbons were detected using a flame ionization detector (FID), and hydrogen was detected using a thermocouple detector (TCD). Calibration curves for CO, $CH_4$, $C_2H_4$, and $C_2H_6$ were plotted using manually injected standards of concentrations ranging from 100 – 1000 ppm. Figures S1-S3 show these graphs and their equations. The peak areas of the ECR products were used to determine concentration in ppm. The concentration of each product obtained was further used to determine the Faradaic efficiencies (%) as described in the main article.

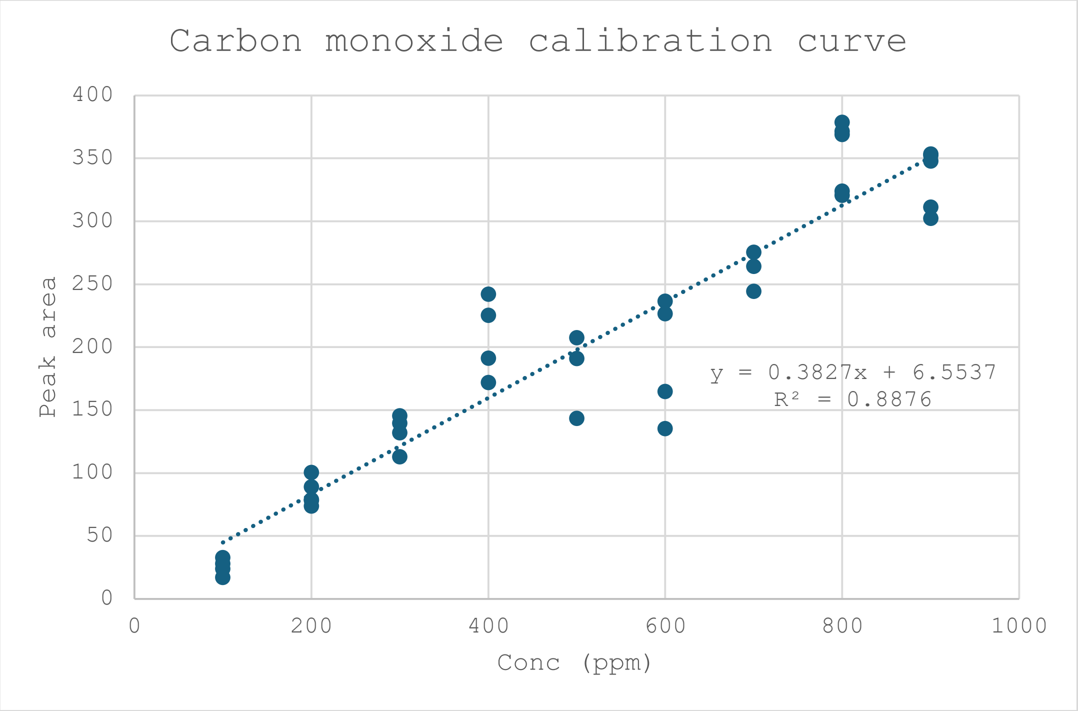


**Figure S2**: Calibration curve of carbon monoxide (CO)

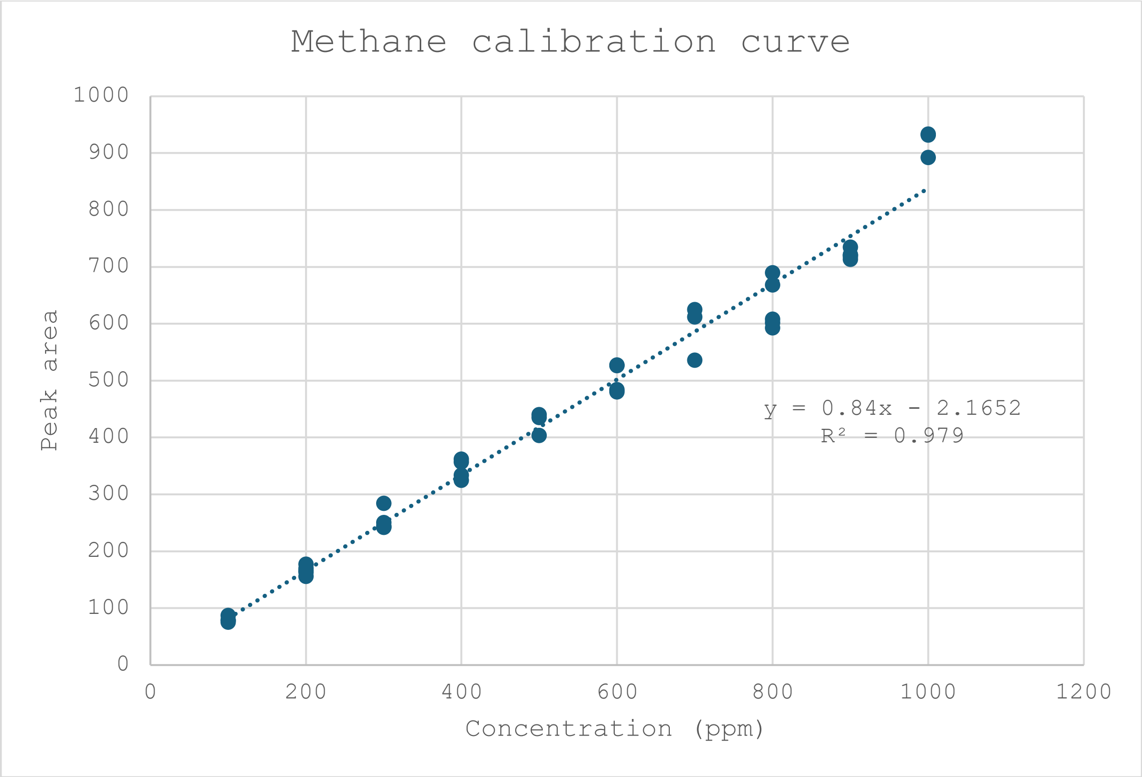


**Figure S3**: Calibration curve of methane ($CH_4$)

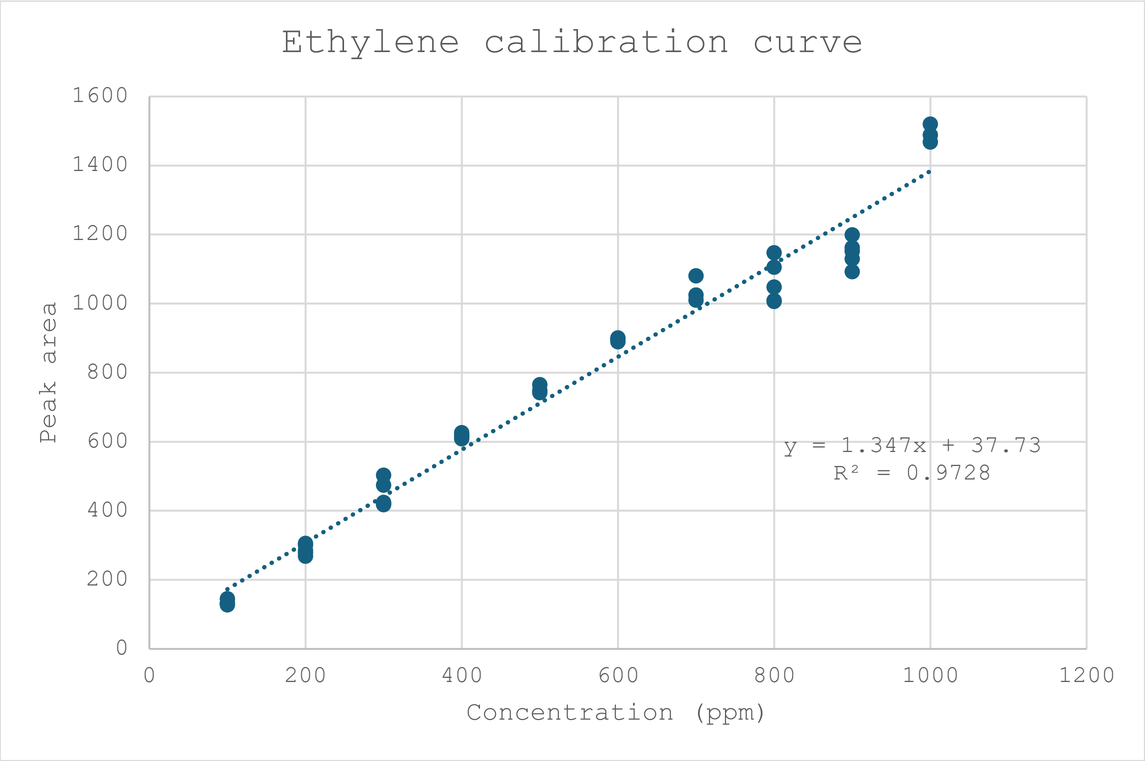


**Figure S4**: Calibration curve of ethylene ($C_2H_4$)

**Table S1.** Summary of ECR using Cu (II) porphyrin based electrocatalysts in literature*.

| Electrocatalyst | Electrolyte | Electrolyser configuration | E vs. RHE ( j ) | Product (FE%) | Reference |
|---|---|---|---|---|---|
| CuP | 0.1 M NaOH | Modified zero-gap PEM water electrolyser | –1.2 V (-202.4 mA/$cm^2$) | CO: 0.103 %<br>$CH_4$: 0.155 %<br>$C_2H_4$: 2.17 % | **This work** |
| PorCu | 0.5M $KHCO_3$ | home-made gas-tight AEM separated two-compartment) | –0.976 V (49 mA/$cm^2$) | $CH_4$: 27%<br>$C_2H_4$: 17% | [40] |
| Cu-PorOH | 0.5M $KHCO_3$ | Two-compartment Nafion-separated Cell. | –1.5 V (23.2 mA/$cm^2$) | $CH_4$: 51.3% | [41] |
| CuTAPP | 1 M KOH | AEM flow cell | –1.63 V (-290.5 mA/$cm^2$) | $CH_4$: 54.8% | [42] |
| CuTPP | 1 M KOH | AEM flow cell | –1.43 V (-60.5 mA/$cm^2$ | $CH_4$: 22.0% | [42] |
| Cu-TDPP-NS | 0.5 M Phosphate buffer solutions (PBS) | AEM flow cell (Three chamber electrolyser configuration) | –1.6 V (-183.0 mA/$cm^2$) | $CH_4$: 70% | [43] |
| Cu-TDPP | 0.5 M Phosphate buffer solutions (PBS) | AEM flow cell (Three chamber electrolyser configuration) | –1.6 V (-107.0 mA/$cm^2$) | $CH_4$: 55.9% | [43] |
| CPF | 0.1 M $KHCO_3$ | H-Cell configuration separated by Nafion® 117 | –1.4 V (-7.5 mA/cm) | $CH_4$: 55.35 %<br>$C_2H_4$: 18.22 % | [44] |

| | | | | | |
|---|---|---|---|---|---|
| CuTCPP-NF | 0.1 M $KHCO_3$ | H-Cell configuration separated by Nafion® 211 | –1.3 V | $CH_4$: 60.1%<br>$C_2H_4$:16.11% | [45] |
| CuTCPP -NS | 0.1 M $KHCO_3$ | H-Cell configuration separated by Nafion® 211 | –1.2 V | $C_2H_4$: 40.6% | [45] |
| CuTBrPP | 1 M KOH | Flow cell (Specialized three-chamber flow electrolyser) | –1.0 V<br>(-173.6 $mA/cm^2$) | $CH_4$: 55.8% | [46] |
| CuT(OMe)PP | 1 M KOH | Flow cell (Specialized three-chamber flow electrolyser) | –1.0 V<br>(-102.7 $mA/cm^2$) | $CH_4$: 33.7% | [46] |
| CuTHPP | 1 M KOH | Flow cell (Specialized three-chamber flow electrolyser) | –1.0 V<br>(-62.9 $mA/cm^2$) | $CH_4$: 22% | [46] |
| CuTPP – Super P | 1 M KOH | Zero-gap Electrolyser with piperION® membrane | ~2.5 V cell voltage | CO: 11%<br>$CH_4$: 6 % | [47] |
| CuTPP - CNT | 1 M KOH | Zero-gap Electrolyser with piperION® membrane | ~3.4 V cell voltage | CO: 0.01%<br>$CH_4$: 0.4 % | [47] |

* *It can be noted from all the citations given in this table that most of the configurations containing Nafion® use a flow cell configuration, and where zero-gap configurations were used, anion exchange membranes were utilized. From this one can understand the uniqueness of the PEM based system and the importance it will provide in the current and upcoming research work on zero-gap* $CO_2$ *electrolyser systems.*

## 3. References


[39] J. Polonský, I.M. Petrushina, E. Christensen, K. Bouzek, C.B. Prag, J.E.T. Andersen, N.J. Bjerrum, Int. J. of Hydrogen Energy, 2012, 37, 2173.

[40] Z. Weng, Y. Wu, M. Wang, J. Jiang, K. Yang, S. Huo, X.-F. Wang, Q. Ma, G.W. Brudvig, V.S. Batista, Y. Liang, Z. Feng, H. Wang, J. Am. Chem. Soc., 2016, 138, 8076.

[41] Y. Zhang, Q. Zhou, P. Wang, Y. Zhao, F. Gong, W.Y. Sun, ChemSusChem, 2022, 15, e202102528.

[42] X.L. Pinger Yu, Q. Wang, H. Huang, W. Weng, C. Peng, Small, 2023, 19, e2205730.

[43] Y.R. Wang, M. Liu, G.K. Gao, Y.L. Yang, R.X. Yang, H.M. Ding, Y. Chen, S.L. Li, Y.Q. Lan, Angew. Chem. Int. Ed., 2021, 60, 21952.

[44] Y. Zhou, S. Chen, S. Xi, Z. Wang, P. Deng, F. Yang, Y. Han, Y. Pang, B.Y. Xia, Cell Rep. Phys. Sci., 2020, 1, 100182.

[45] M. Sun, Z. Tao, X. Xu, S. Min, L. Kang, Appl. Catal. A Gen., 2023, 666, 119406.

[46] H. Jiang, P. Zhao, H. Shen, S. Yang, R. Gao, Y. Guo, Y. Cao, Q. Zhang, H. Zhang, Small, 2024, 20, 2304998.

[47] W. Wiesner, J.Y.M. Arias, J. Jökel, R. Cao, U-P. Apfel, Chem. Commun., 2024, 60, 14668.
